# Dr. William S. Playfair

**Published:** 1903-09

**Authors:** 


Dr. William S. Playfair, of London, died at St. Andrews, Scotland, August 13, 1903, aged 67 years. He was professor of obstetric medicine in King's College for 25 years, was the author of a treatise on obstetrics and was regarded as one of the most distinguished obstetricians of his time. He also wrote several books and monographs on gynecology and other subjects. Dr. Playfair was married in 1864 to Miss Emily Kitson, and they had one son and three daughters.

